# The use of machine learning to predict somatic cell count status in dairy cows post-calving

**DOI:** 10.3389/fvets.2023.1297750

**Published:** 2023-12-08

**Authors:** Jake S. Thompson, Martin J. Green, Robert Hyde, Andrew J. Bradley, Luke O’Grady

**Affiliations:** ^1^School of Veterinary Medicine and Science, University of Nottingham, Nottingham, United Kingdom; ^2^Quality Milk Management Services Ltd., Easton Hill, United Kingdom

**Keywords:** dairy cow, mastitis, dry period, machine learning, probabilistic predictions

## Abstract

Udder health remains a priority for the global dairy industry to reduce pain, economic losses, and antibiotic usage. The dry period is a critical time for the prevention of new intra-mammary infections and it provides a point for curing existing intra-mammary infections. Given the wealth of udder health data commonly generated through routine milk recording and the importance of udder health to the productivity and longevity of individual cows, an opportunity exists to extract greater value from cow-level data to undertake risk-based decision-making. The aim of this research was to construct a machine learning model, using routinely collected farm data, to make probabilistic predictions at drying off for an individual cow’s risk of a raised somatic cell count (hence intra-mammary infection) post-calving. Anonymized data were obtained as a large convenience sample from 108 UK dairy herds that undertook regular milk recording. The outcome measure evaluated was the presence of a raised somatic cell count in the 30 days post-calving in this observational study. Using a 56-farm training dataset, machine learning analysis was performed using the extreme gradient boosting decision tree algorithm, *XGBoost*. External validation was undertaken on a separate 28-farm test dataset. Statistical assessment to evaluate model performance using the external dataset returned calibration plots, a Scaled Brier Score of 0.095, and a Mean Absolute Calibration Error of 0.009. Test dataset model calibration performance indicated that the probability of a raised somatic cell count post-calving was well differentiated across probabilities to allow an end user to apply group-level risk decisions. Herd-level new intra-mammary infection rate during the dry period was a key driver of the probability that a cow had a raised SCC post-calving, highlighting the importance of optimizing environmental hygiene conditions. In conclusion, this research has determined that probabilistic classification of the risk of a raised SCC in the 30 days post-calving is achievable with a high degree of certainty, using routinely collected data. These predicted probabilities provide the opportunity for farmers to undertake risk decision-making by grouping cows based on their probabilities and optimizing management strategies for individual cows immediately after calving, according to their likelihood of intra-mammary infection.

## Introduction

The prevention of mastitis remains a priority for the global dairy industry since it has serious welfare and economic ramifications (1–3). In addition, despite a reduction in overall use of clinical mastitis and antibiotic use across the dairy sector, dry cow therapy treatments remain the largest contributors to the industry’s antibiotic usage (2, 4, 5). Preventive strategies for mastitis are often formulated from herd epidemiological patterns of udder health, defined using assessment of clinical mastitis events and routine somatic cell count data (6). The dry period is a critical time for udder health (7–10) because it represents a high-risk time for new intra-mammary infections (IMI) (11, 12) and a time to cure existing IMIs (13). As day-to-day electronic on-farm recording intensifies, so have the opportunities to conduct real-time predictive analyses for individual cows increased. Predicting on-farm outcomes using complex methods such as machine learning includes sub-clinical and clinical mastitis at the individual level (14–22), evaluating udder infection status at dry-off to inform selective dry cow therapy decisions (SDCT) (23, 24), and informing mastitis pattern diagnosis at a herd level (17, 25). Supervised machine learning methods provide new ways to process data and provide supportive information to farmers.

Given the wealth of udder health data commonly generated through routine milk recording and the importance of udder health to the productivity and longevity of individual cows (1, 7, 26), an opportunity exists to extract greater value from cow-level data to inform cow- and group-based decision-making based on the risk of intramammary infection. Machine learning methods have been explored to predict IMI status at subsequent milk recording in both dairy cows (21) and buffaloes alongside climate data (22). These analyses were undertaken on small datasets and not externally validated to assess generalizability for external farms. Attempts have been made to utilize regularly recorded farm data to infer risk factors associated with failure to cure and prediction of new IMI over the dry period using mixed effects models, but the performance of these models was not externally validated to understand generalizability (27). There have been a number of meta-analyses/reviews in the area of predicting cure and new IMI outcomes dependent on dry cow therapy; however, this research does not include variables based on cow udder health histories to obtain predictions (24, 28, 29). This research primarily focused on using such data to inform selective dry cow therapy treatment decisions rather than assess the probability risk of having an intra-mammary infection post-calving irrespective of treatment protocols. Furthermore, the imbalance of the outcomes in these datasets has led to the reporting of exaggerated accuracies without assessment of sensitivity or specificity, with at least one of these metrics being poor. In addition, an assessment of the calibration fit of the probabilities across individual cows was not undertaken. This is important as it provides greater context into the ability of a model to categorize individuals into groups of risk. If a model calibrates well, then higher-risk groups of animals can be identified and managed appropriately with the aim of reducing this risk in a targeted method. This means that an accurate prediction model of individual cow infection risk post-calving has not yet been established. The use of large-scale external datasets to validate model performance in terms of calibration fit is critical to understanding how these models will perform when provided with data from new farms (30, 31). This helps to ensure the generalizability of predictive models and was a focus of this research.

Cows with a high probability of an IMI post-calving can be managed with additional care to minimize the likelihood of transmission of infection of herd-mates, for example, by implementing additional hygienic procedures at milking for these cows. The use of machine learning algorithms to interpret the “big data” collected on the farm has the potential to identify these higher-risk cows using probability estimates for individual cows using real-time data. The assessment of calibration performance can ensure generalizability to cows and herds outside of train-test datasets. Optimized calibration performance places emphasis on managing the risk of groups when the accuracy performance of predicting individual outcomes is difficult. This provides an opportunity to create value from the data collected on farm that can be used for day-to-day management purposes when identifying cows that are at a lower risk of calving into a subsequent lactation as uninfected.

The aim of this research was to construct a machine learning model, using routinely collected farm data, to make probabilistic predictions at drying off of a cow’s risk of an intra-mammary infection post-calving.

## Materials and methods

Ethical approval for this observational study was received through the University of Nottingham ethical review process, number: 3114 200,218, and Innovate UK project number: 107111.

### Data source

Anonymized data were obtained as a convenience sample from 108 herds that undertook regular milk recording analysis using a single milk recording laboratory (QMMS Ltd.) from 1990 to 2022. The data available included anonymized farm and cow identifiers, parity, dry-off and calving dates, milk recording dates, somatic cell counts, milk parameters (yield / fat %/ protein %), and clinical mastitis event records.

### Data cleaning, processing, and descriptive analysis

All data processing and analysis were undertaken in R-statistical software version 4.1.3 (32), with the addition of the following packages *Tidyverse* (33), *Caret* (34), *PresenceAbsence* (35, 36), *XGBoost* (37), and *SHAPforxgboost* (38, 39).

**Figure 1:**
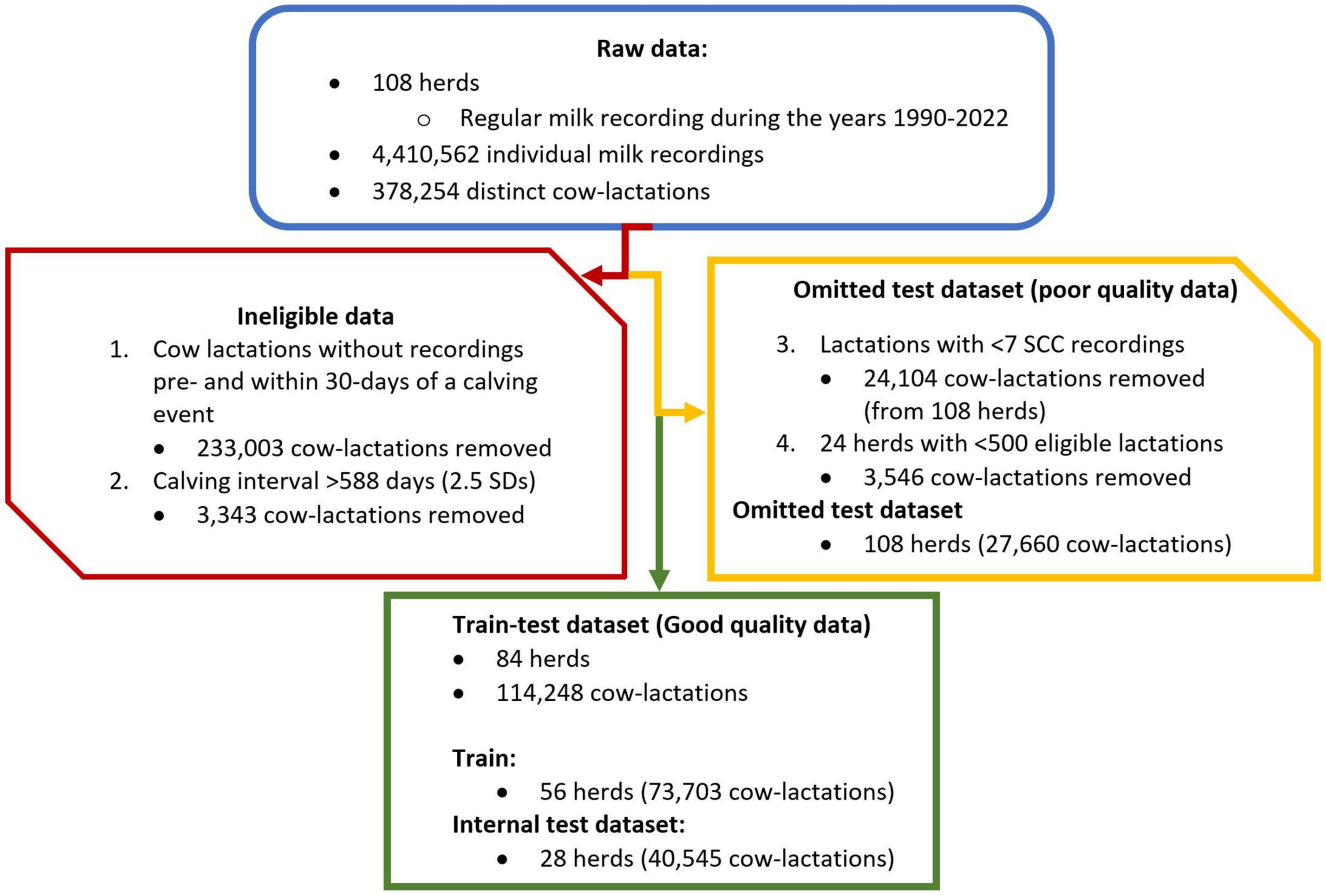
Flowchart to show the data-cleaning process. The process starts with raw data (blue/curved triangle), then steps one and two permanently remove data (red/hexagon). Data are removed in steps three and four but retained separately to be used as a poor-quality test dataset (yellow/ pentagon). This provided the final good-quality dataset (green/ rectangle) which was then split at herd level (2:1) to create a 56-herd train dataset and a 28-herd internal test dataset.

Data were explored through visualization to examine the distributions and correlation of variables, and to identify missing or erroneous data. Data was cleaned and split into train and test datasets as shown by the flowchart in Figure 1. Firstly, cow-lactations were removed where milk recording data were not available in a previous lactation or within 30 days post-calving event; this excluded maiden heifers (no previous lactation) and multiparous cows with missing data. Duplicate data, milk recordings with missing SCC and protein % and fat % data, and records where a cow’s calving interval was calculated to be greater than 2.5 standard deviations from the mean were removed from the dataset. For the main dataset, cow records were also removed if the lactation prior to a dry period contained fewer than seven somatic cell count recordings, and herds were removed if they provided fewer than 500 cow-lactations. Of the 108 herds available, 84 contained data that met the pre-determined inclusion criteria and were therefore included in the main dataset and used to construct and test machine learning models. The remaining 24 herds with inferior data were used in an additional external model validation procedure, as described below.

Although no perfect threshold of SCC exists to define an IMI, to account for increased SCC levels in uninfected cows in the first days after calving (40), a raised SCC was defined as being ≥400,000 cells/mL for samples collected 1–4 days post-calving and ≥ 200,000 cells/mL for samples collected 5–30 days post-calving. In addition, any cow with clinical mastitis recorded within 30 days of calving was deemed to have an IMI attributable to the dry period (12) and was therefore classified as infected, which is the equivalent of a raised SCC. These definitions have been used to align with the main UK milk recording analytical software (41) and used as part of the national UK Dairy Mastitis Control Plan (DMCP) scheme (42, 43).

The outcome variable of interest was the individual cow somatic cell count status (as a proxy for intra-mammary infection) in the first 30 days after calving; each cow was either defined as having a raised SCC or not in this post-calving period. This was based on the definition of a raised SCC as defined earlier.

Data distributions to assess farm-level descriptive statistics were calculated to compare variability between train and test datasets. These included:

- Unique cow-lactation contributions to the train-test datasets: The total number of eligible cow-lactations available for input into the model per farm. This could include multiple lactations per cow if the data was available.
- Predicted 305-day yield per farm: the sum of the average predicted 305-day milk yield curve (44) was calculated per farm from all milk recordings available.
- New dry period intra-mammary infection rate: Calculated as the number of cows that calved in with a raised SCC (as defined above) divided by the number of animals defined as uninfected prior to dry-off (based on an SCC within 60 days of a dry-off event being less than 200,000 cells/mL).
- Cow level mean SCC per lactation per farm: The mean of the somatic cell counts for an individual cow lactation was calculated, then the mean of these was calculated per farm, and this distribution was assessed.

### Models to predict the probability of a raised somatic cell count post-calving: model selection, parameter tuning, and evaluation of performance

The final cleaned dataset was randomly split into train (56 farms) and test (28 farms) datasets. The training dataset was used to develop and tune machine learning models and the separate test dataset was used to assess external model validity (31). A second test dataset was created using the data removed prior to obtaining the final cleaned dataset, hereto named “omitted dataset,” which was used to assess model performance on poor-quality datasets.

Based on biological plausibility, predictor variables were engineered that were deemed to be of potential importance for the prediction of SCC status post-calving. Definitions of the new variables are provided in Table 1. During the training phase, all variables were used (variables used in the final model are annotated by † in Table 1).

**Table 1:** Predictor variables, with definitions, tested in the models of dry period infection status.

Variable	Definition
^†^Parity	Parity of the lactation prior to dry-off period
Dry-off IMI status	Binomial parameter stating whether the udder was classed as infected or uninfected at dry-off. An infected status pre-dry-off was based on a cow’s last SCC of the lactation (within 60 days of a dry-off event) being greater than 200,000 cells/mL, or if the cow was missing from a recording then based on a recording of clinical mastitis within 7 days of the milk recording.
^†^Calving interval	Time in days between a calving event recorded in the new lactation and previous lactation
Lactation length	The time between the recording of a calving event and a dry-off event
Month of dry-off	The month of the recorded dry-off date
Month of calving	The month of the calving date for the subsequent lactation
^†^First SCC*	The first recorded SCC in the lactation prior to dry-off event
Consecutive SCC >200*	The categorical variable which states whether there had been three consecutive SCCs >200 in the lactation prior to dry-off event (TRUE/FALSE)
^†^Median SCC*	The median SCC of the recorded SCC in the lactation prior to dry-off event
Mean SCC*	The mean SCC of all records in the lactation prior to dry-off event
^†^Min SCC	The lowest recorded SCC in the lactation prior to dry-off event
Max SCC	The highest recorded SCC in the lactation prior to dry-off event
^†^Mean first 3 SCC	The mean SCC of the first three recordings in the lactation prior to dry-off event
Mean last 3 SCC	The mean SCC of the last three recordings in the lactation prior to dry-off event
Ratio of mean first 3 to last 3 recordings	The mean SCC of the last three recordings in the lactation prior to dry-off event divided by the mean SCC of the first three recordings in the lactation prior to dry-off event
^†^Last SCC	The last recorded SCC in the lactation prior to a dry-off event
^†^%SCC < 50	The percentage of SCCs, in the lactation prior to dry-off event, less than 50 k cells divided by the total number of recordings
^†^%SCC > 100	The percentage of SCCs, in the lactation prior to dry-off event, greater than 100 k cells divided by the total number of recordings
%SCC > 200	The percentage of SCCs, in the lactation prior to dry-off event, greater than 200 k cells divided by the total number of recordings
%SCC > 400	The percentage of SCCs, in the lactation greater prior to dry-off event, than 400 k cells divided by the total number of recordings
%SCC > 1,000	The percentage of SCCs, in the lactation prior to dry-off event, greater than 1 million cells divided by the total number of recordings
Peak yield	The highest daily yield recorded in the lactation prior to dry-off event
Yield at last recording	The final yield recorded prior to a dry-off event
Max fat	The highest milk fat percentage recorded in the lactation prior to dry-off event
Max protein	The highest protein percentage recorded in the lactation prior to dry-off event
Min fat	The lowest milk fat percentage recorded in the lactation prior to dry-off event
Min protein	The lowest protein percentage recorded in the lactation prior to dry-off event
Mean fat	The mean milk fat percentage recorded in the lactation prior to dry-off event
Mean protein	The mean protein percentage recorded in the lactation prior to dry-off event
^†^New IMI 12-month rate	The herd level new dry period IMI rate, as calculated by the total number of new DPIMIs in the previous 12 months’ data prior to the cow’s calving month divided by the total number of individuals in the eligible population of non-infected at the time of dry-off
^†^New IMI 6-month rate	As above, but for the previous 6 months’ data prior to the cow’s calving date

^†^Indicates used in the final model.

Following initial exploration with a variety of algorithms, a gradient-boosting machine learning decision tree algorithm was chosen using *XGBoost* (37). This algorithm is a supervised decision tree algorithm that combines ensemble learning and gradient-boosting techniques. The method iteratively trains an ensemble of shallow decision trees to additively improve the model’s performance with the goal of minimizing the error. Due to these characteristics, it was deemed to be the most appropriate model to handle the multicollinearity in the dataset. Ten-fold cross-validation was used to tune the model learning rate parameter(eta). Due to the imbalance of the observed outcome measure in the dataset, tuning based on raw accuracy was considered inappropriate (45, 46). Model tuning parameters were analyzed based on the cross-validation results of the training dataset. Other model tuning parameters were set as follows: subsample = 1, max depth = 4, columns sampled by tree = 1, and number of parallel trees = 1 (37, 47).

The final model included the top 11 that were variables judged by relative importance and Shapley Additive exPlanations (SHAP) values. The SHAP feature value provides a proxy effect measurement for each variable based on the data dispersion for each variable and contribution toward the prediction output. To assess performance based on the number of variables included, the methods above were repeated using combinations of the variables classed as the highest on variable importance from 1 to 15. Model complexity was limited through variable selection assessment using SHAP values (39) and variable relative importance values. The model with the top 11 variables only was deemed closest in performance to the all-variable model, while reducing the complexity with a number of variables present. These variables were selected based on having a variable importance greater than 0.7 and biologically important SHAP values which contributed to the prediction outputs. Therefore, the model containing 11 variables was taken forward (48, 49).

The final model performance was evaluated from predictive outcomes using the external test dataset. Such external validation was considered the gold standard for assessing the performance of machine learning models (31). A confusion matrix was created to assess observed versus predicted outcomes. Discrimination statistics were produced against a threshold of 0.5 and assessed against accuracy, balanced accuracy [calculated by (Sensitivity + Specificity)/2], positive predictive value (PPV), and negative predicted value (NPV). A calibration plot where model-predicted probabilities were binned into groups of 10 and compared to observed outcomes was used to assess external model validity. Calibration plot fit was assessed using visual assessment, Scaler Brier Score, and Mean Absolute Calibration Error (MACE) (50). MACE assesses the level of predictive error by averaging the absolute values of deviance; the lower this value the lower the error within the predictions of the model. This feature was also calculated on a per-farm basis for the test dataset to assess model calibration performance across the individual farms. Brier scores use the average squared difference between an outcome and predicted probability to determine the model fit for categorical variables (50, 51). A scaled Brier score fixes this outcome to between zero and one. The closer to zero the better the goodness-of-fit and these parameters can be assessed similarly to an R^2^ for linear predictions.

### Incomplete data set testing

A final assessment of external model validity was undertaken on data that were previously omitted from the train-test datasets. This included cow-lactations with fewer than seven milk recordings and farms that had data with fewer than 500 complete cow-lactations. Assessment of model performance was undertaken as described above.

## Results

### Descriptive analysis

The dataset from the 108 herds that undertook regular milk recording analysis using a single milk recording laboratory (QMMS Ltd.) from 1990 to 2022 containing a total of 4,410,562 milk recording events were available from 378,254 individual cow-lactations prior to data-cleaning. The number of cow-lactations removed during cleaning were:

- Cow lactation without a recording pre or within 30 days of a calving event: *n* = 233,003 lactation removed.
- Had a calving interval of greater than 588 days (2.5sd from the mean): *n* = 3,343 lactations removed.
- Contained fewer than seven milk recordings in the previous lactation: *n* = 24,104 lactations removed.
- From a herd with fewer than 500 (first interquartile) cow-lactations worth of results: 24 farms removed; *n* = 3,546 lactations.

This resulted in a total of 114,248 cow-lactations from 84 farms being eligible for the train and test datasets. Data populations of train and test datasets were compared and they showed that farm-level parameters were similar, as shown in Table 2. For the training dataset, unique cow-lactation contributions ranged from 262 to 2,709 cows per farm with a mean of 908, 305-day yield ranged from 5,641–12,803 L with a mean of 9,180 L, and new dry period intra-mammary infection rate varied between 0.062 and 0.299 with a mean of 0.154. Cow level mean SCC per lactation per farm ranged from 87 to 361, with a median of 195. The test dataset showed similar variability in these farm-level parameters; unique cow contributions ranged from 267 to 2,815 cows per farm with a mean of 1,084, 305-day yield ranged from 5,093 to 12,015 L with a mean of 8,694 L, and new dry period intra-mammary infection rate varied between 0.058 and 0.296 with a mean of 0.156. The cow level mean SCC per lactation per farm ranged from 79 to 407, with a median of 208.

**Table 2:** Summary of farm herd-level details for each dataset evaluated.

		Farm-level parameters			
Dataset	Summary statistic (at farm level)	Unique cow-lactation contributions per farm	Predicted 305-day yield per farm	Cow level mean SCC per lactation per farm	Proportion of all lactations with new dry period intra-mammary infection rate per farm
Train farms (*n* = 56)	Min	262	5,641	87	0.062
	Median	725	9,156	195	0.147
	Mean	908	9,180	207	0.154
	IQR	576	2,354	99	0.069
	Max	2,709	12,803	361	0.299
Test farms (*n* = 28)	Min	267	5,093	79	0.058
	Median	911	8,874	208	0.151
	Mean	1,084	8,694	215	0.156
	IQR	845	2,336	77	0.045
	Max	2,815	12,015	407	0.296
Omitted farms* (*n* = 24)	Min	53	5,533	3	0.000
	Median	360	7,840	214	0.147
	Mean	373	7,830	231.7	0.144
	IQR	311	1,231	105	0.086
	Max	1,110	11,966	574	0.316

Described are summary statistics for the number of cows present in each herd’s data, the predicted 305-day yield for the herd, the raw SCC data, and the herd’s new dry-period intra-mammary infection rate across the recording period. *Data that were previously omitted from the train-test datasets. This included cow-lactations with fewer than seven milk recordings and farms that had data with fewer than 500 complete cow-lactations.

### Model of the probability of intra-mammary infection after a dry period

#### The dataset

The final dataset of 84 farms was split into a 56-farm training and a 28-farm test dataset. The training dataset contained 73,703 cow-lactation outcomes, with 12,695 (17.2%) classed as having a raised SCC post-calving and 61,008 (82.8%) classed as not having a raised SCC. Within this training dataset group, 52,933 (71.8%) of the cow-lactations were classed as uninfected (based on an SCC within 60 days of a dry-off event being less than 200,000 cells/mL) at the point of dry-off with the remaining 20,770 classed as having an intra-mammary infection (based on an SCC within 60 days of a dry-off event being greater than 200,000 cells/mL, or if the cow was missing from a recording then based on a recording of clinical mastitis within 7 days of the milk recording). The test dataset of 28 farms contained 40,545 cow lactation outcomes; of these, 33,529 (82.7%) dry periods resulted in being classed as uninfected and 7,016 as infected. Prior to dry-off, 29,435 (72.6%) of the lactations were classed as uninfected with the remaining 11,110 lactations classed as infected based on the parameters described earlier in the section.

#### Variable selection

**Figure 2:**
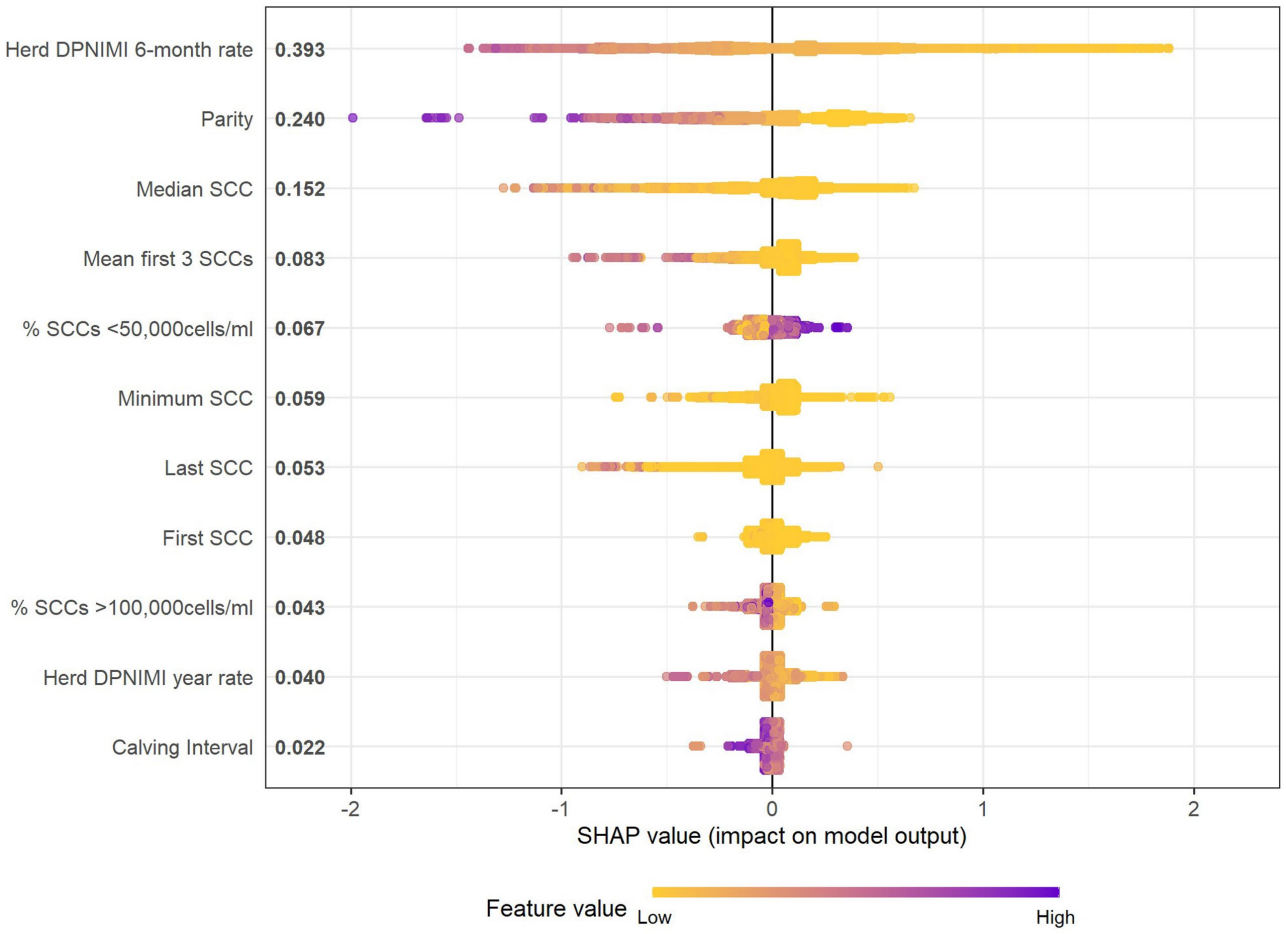
SHAP summary beeswarm plot which details the variable names in order of importance based on mean SHAP value (y-axis) and SHAP values or change in log-odds of being uninfected (x-axis), and gradient color represents the raw data value of the variable (color) of each of the 11 predictor variables (y-axis) within the 56-farm training dataset of the final XGBoost model to predict the probability of intra-mammary infection post-calving. DPNIMI, dry period new intra-mammary infection; SCC, Somatic cell count (refer to lactation prior to outcome).

The final model included the top 11 variables judged by relative importance (Table 3) and Shapley Additive exPlanations (SHAP) values (Figure 2). Using internal-fit cross-validation, this model performed with an accuracy of 83.1%, a balanced accuracy of 52.8%, a sensitivity of 99.0%, a specificity of 6.6%, a PPV of 83.6%, and an NPV of 58.3%. SHAP helps to obtain a measure for the impact of a variable when interpreting black-box models, while accounting for the interactions between all variables tested. In the final model, the three most influential variables were herd new dry period intra-mammary infection rate over the previous 6-months (SHAP = 0.393), parity (SHAP = 0.240), and median SCC of previous lactation (SHAP = 0.152). For example, herd new dry period IMI 6-month rate as a variable shown in Figure 2 has a distinct color change across the SHAP value showing data dispersion as well as contribution toward the prediction output (Figure 2). High rate values (purple) are linked to a decreased probability of being non-infected after the dry period and lower rate values (yellow) are linked with an increased probability of being non-infected after the dry period. When using parity, as an example, the SHAP plot shows a trend that higher parities (purple) are associated with larger negative SHAP values (toward the left-hand side of the plot). This indicates that increased parity is linked with a lower probability of being uninfected within the first 30 days post-calving event.

**Table 3:** The 11 variables used in the final XGBoost model (with relative importance) to predict the probability of intra-mammary infection post-calving.

Variable	Relative importance
Herd new dry period IMI 6-month rate	38.15
Median SCC of previous lactation	28.06
Parity	13.82
Percentage of SCCs less than 50 in previous lactation	4.29
Mean of first three SCCs of previous lactation	4.09
Last SCC of previous lactation	4.08
Minimum SCC of previous lactation	2.70
Herd new dry period IMI year rate	1.94
Percentage of SCCs greater than 100 in previous lactation	1.42
Calving interval	0.73
First SCC of previous lactation	0.72

#### External model validity

**Figure 3:**
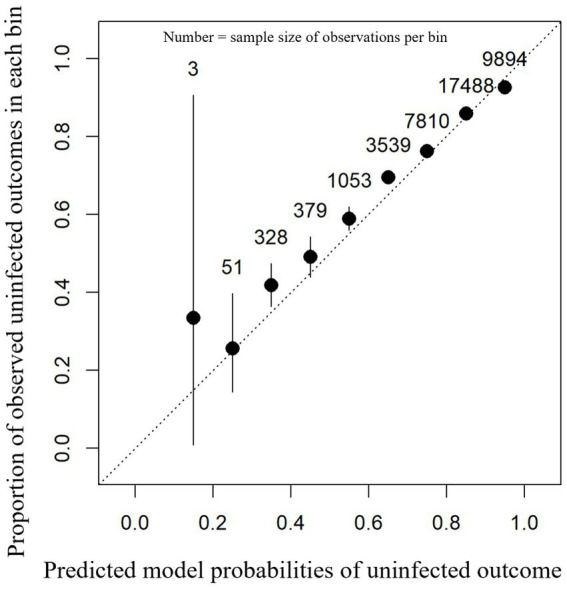
Calibration plot showing the proportion of observed uninfected outcomes (y-axis) against each 0.1 bin (between 0 and 1) of the model-predicted probabilities for intra-mammary infection status after a dry period (x-axis). This contains the predictions for each cow-lactation from the 28-farm external dataset based on an XGBoost model. 95% confidence intervals (black lines) are shown around the mean proportion (black dot) for each bin.

For the external test dataset, the model calibration was of high performance as shown in Figure 3. An accuracy of 82.9% was observed in the external farm test dataset with a balanced accuracy of 52.5%, sensitivity of 99.0%, specificity of 6.0%, PPV of 83.5%, and NPV of 58.2%. Statistical assessment of calibration showed a scaled Brier score of 0.095 and a MACE of 0.0088. From the calibration plot (Figure 3), it can be seen that the majority of predicted probabilities for having a normal SCC status post-calving were between 60 and 90%.

For each test farm, a MACE was calculated; the median farm had a MACE of 0.030 with an interquartile range between 0.026 and 0.037 (mean = 0.033/minimum = 0.012/maximum = 0.057). These data show that model performance based on MACE was similar for each farm in the test dataset.

#### Complete versus incomplete data comparisons

The model was further tested on data that were omitted from the train and test datasets. This resulted in the use of data from all 108 farms, as the data from the 24 farms which were omitted due to the small sample size (*n* ≤ 500 cow-lactations) were combined with the poor data from the 84 train-test farms. This new dataset included cow-lactations from farms with fewer than 500 cow-lactations (*n* = 3,546 cow-lactations) and from cow-lactations with fewer than seven SCC recordings (*n* = 24,104 cow-lactations). A total of 27,660 new cow-lactations were included from across all 108 original farms, and 22,982 (83.1%) rows were from dry periods where cows were classed as uninfected with the remaining 4,678 classed as having an intra-mammary infection. Of the cow-lactations in this dataset, 21,229 (76.7%) cow-lactations were identified as uninfected prior to dry-off, with the remaining 6,431 classed as infected.

**Figure 4:**
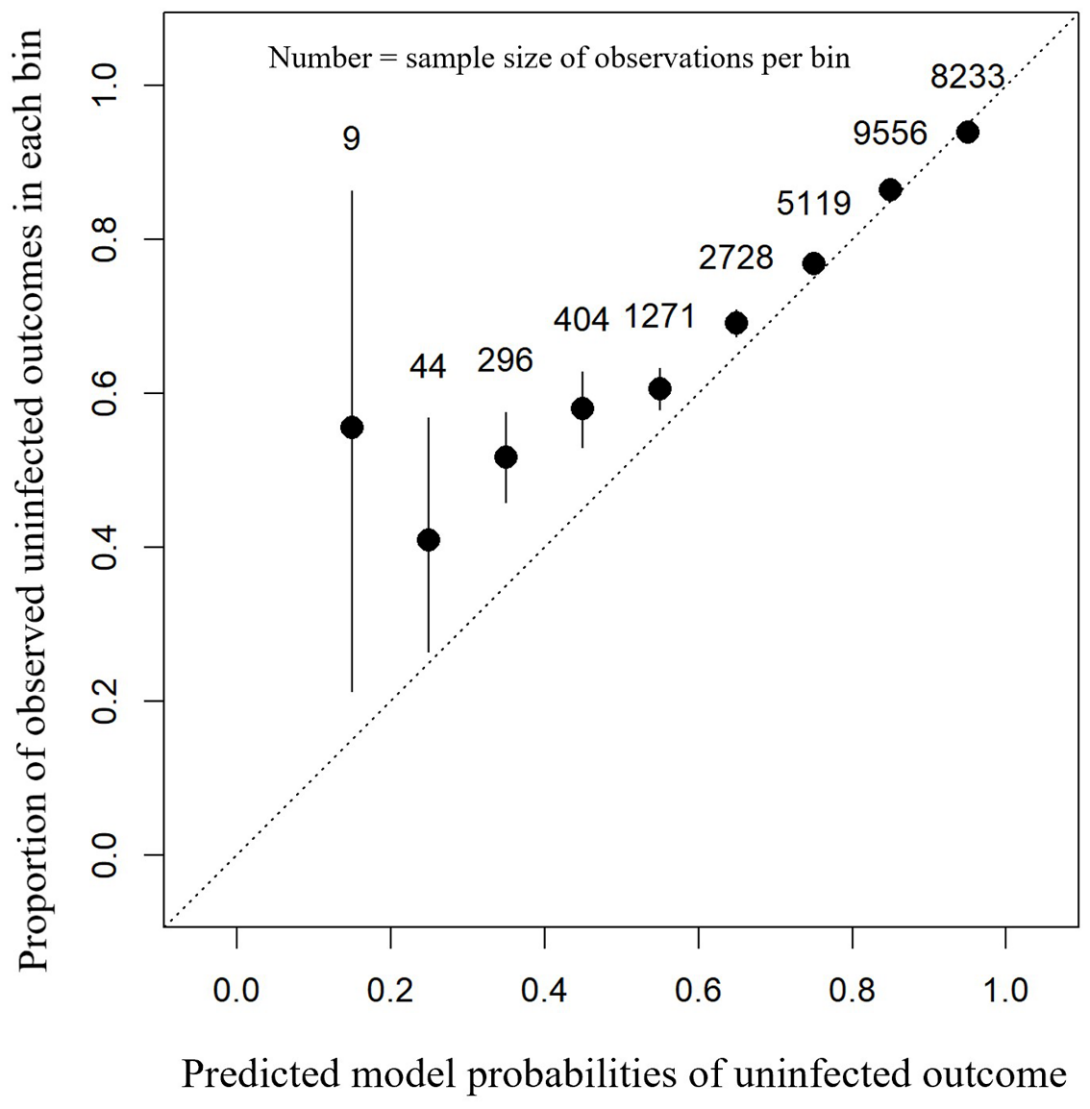
Calibration plot showing the proportion of observed uninfected outcomes (y-axis) against each 0.1 bin (between 0 and 1) of the model-predicted probabilities for intra-mammary infection status after a dry period (x-axis). This contains the predictions for each cow-lactation from the omitted cow-lactations dataset based on an XGBoost model. 95% confidence intervals (black lines) are shown around the mean proportion (black dot) for each bin.

For this dataset, a balanced accuracy of 52.8%, accuracy of 82.9%, sensitivity of 98.2%, specificity of 7.3%, PPV of 83.9%, and NPV of 45.6% were achieved. The calibration curve of model performance with these data was good (Figure 4). Assessment of calibration curve fit showed a scaled Brier score of 0.130 and a MACE of 0.016. Unlike the test dataset, the model appeared to perform less well for the lower observed probabilities (<50%) with the calibration curve of predicted probabilities being lower than observed probabilities (Figure 4).

## Discussion

The aim of this research was to predict individual cow probabilities of a low SCC in the 30 days post-calving. Our results revealed that this was achievable with an acceptably low calibration error in an external dataset, suggesting that such a predictive model has the potential to be of practical value in a commercial setting. The majority of predictions ranged between 60 and 90% for cows to remain uninfected during the first 30 days in milk following a dry period; however, cows at relatively high risk could be differentiated effectively from those of lower risks.

The model’s ability to distinguish groups of animals according to risk is novel and has the potential to be an effective on-farm tool to aid farmers in identifying cows that require an adjusted management regime. The output probabilities mean that animals could be placed into probabilistic subsets of high, medium, or low-risk categories. For the higher-risk animals, targeted management practices could be implemented to minimize the risk of subsequent pathogen transmission between cows. For example, after calving, high-risk cows could be managed in a separate group, milked last through the parlor, or could be selectively checked with a California Milk Test early in lactation to confirm a raised SCC. Alternatively, high-risk cows could be managed within the main herd but with increased hygiene procedures (such as additional disinfection) put in place when they are milked. Dairy farming has become linked to big data outputs, and examples include sensor and robotic milking data (52). Farmers can now use such data for on-farm decision-making, for example, estrous detection, but not all outputs have been integrated on a routine basis (53). By providing probabilistic options for decision-making, it is likely farmers and advisors will be able to make more effective use of data. However, despite the availability of data, a “data divide” exists among dairy farmer with barriers remaining that limit the use of such outputs, which includes skillset, wanting to spend time outdoors rather than in office environments, and access to discussion groups or veterinarians to validate interpretation (54). The latter has been found to be a key driver in decision-making for changing management practices given the importance of social referents, especially for interpretation of on-farm data (55).

The herd mean new dry period IMI rate over the previous 6 months was the most influential variable in our final predictive model. This suggests that management during the dry period is the key area that determines udder health status after calving and this has been reported previously (7, 9, 10, 56). This information could be used to promote targeted management of cows during the dry period. The strategies to help reduce the risk to more susceptible individuals within a herd could be, for example, greater space allowance or increased frequency of bedding material application. It should also be noted that these changes may be more challenging for smaller herds.

The three historic individual cows’ SCC parameters that were useful in the prediction of post-calving SCC were from the previous lactation: median SCC, mean of the first three SCCs, and the percentage of SCCs less than 50,000 cells/mL. The probability of calving into a subsequent lactation with a low SCC decreased as the median SCC and mean of the first three SCCs of the previous lactation increased. There is biological plausibility regarding these SCC variables with increased risk of a high SCC post-calving as previous research suggests that cows with a high SCC in a previous lactation are either more susceptible to new infections or an existing infection fails to cure across the dry period (57). The percentage of SCCs less than 50,000 cells/mL parameter showed a bimodal trend with cows that showed either a high or low percentage in this variable having a decreased probability of calving into a subsequent lactation uninfected. This bimodal pattern of increased risk of intra-mammary infection has been hypothesized before with high and low SCCs, with potential links described to an increased risk of clinical mastitis (58). Given SCC parameters have the potential to be correlated, the method of using XGBoost, a decision tree method, is appropriate as the performance of these models remains stable with datasets that contain multicollinearity. This is because it independently splits trees without prior knowledge of importance, thus making it a robust method when using datasets with potential for highly correlated variables (59). The aim of this research was for prediction rather than inference, meaning that the multicollinearity of variables is not of concern when assessing the performance of the model. However, it is worth noting that this also means that the variables selected for the final model are due to prediction performance and the variables selected or omitted should not be identified as having (or not) a definitive biological impact on a raised SCC in the first 30 days post-calving.

Variables incorporated in our final prediction model had similarities to those identified in previous inferential research using cow lifetime records that investigated IMI status across the dry period (30). That study identified similar but not identical covariates to our model such as parity, proportion of SCC recordings >199,000 cells/mL, SCC on the last test day, and lactation length. However, model-predicted probabilities of infection status were not reported in that research and it is therefore difficult to compare model performance.

External validation is an essential though under-used technique to evaluate the possible generalizability of inferential and predictive models (31, 60). Our external validation provided strong evidence that the model is likely to be generalizable to herds similar to those used in this research (Table 2), and it was particularly notable that model performance remained good even when data quality used in external validation was reduced. However, for the reduced dataset with missing data, model performance was slightly poorer for the lower predicted probability values (<50% of remaining uninfected post-calving). This is likely to be due to a reduced signal being provided by poorer-quality data features and emphasizes the importance of high-quality data when implementing such decision-support tools on-farm. Nonetheless, our results indicate that patterns remained sufficiently identifiable in this poor-quality dataset for safe predictions to be made and suggest that the model could continue to perform well even as data availability decreases.

Data used in this study were collected over a long time period, 1990–2022, during which time farm management and SCC analysis methods may have changed. It is unknown whether these factors could have an impact on model performance for particular time periods, but sample size, data quality, and model calibration performance measures suggest this was unlikely to have influenced the final model.

In this study, the dry period remains a time where data is not commonly recorded but is a key driver of udder health post-calving. One limitation of this lack of dry period-specific data was that dry cow antimicrobial therapy status was unknown for all animals in the analysis and the knowledge of treatment could have further improved the predictive performance of the final model. Similarly, the use of internal teat sealant was also unspecified, and this is also known to be effective in reducing herd dry period new infection rates (61–63). Future research could incorporate dry cow therapy treatments into a machine learning algorithm which may refine predictive probabilities of infection status post-calving. Despite this lack of knowledge of dry cow treatments, our model performed well; the model was still able to discriminate between cows at higher and lower risks of a raised SCC after calving. As well as information on dry cow treatments, prediction of SCC status post-calving may be improved with data on herd-level dry period management factors and further cow-level information outside general milk recording information. Other variables that could be of value in prediction include udder conformation and cow genotype.

## Data availability statement

The data analyzed in this study is subject to the following licenses/restrictions: not available due to commercial sensitivity. Requests to access these datasets should be directed to JT, jake.thompson2@nottingham.ac.uk.

## Author contributions

JT: Conceptualization, Data curation, Formal analysis, Investigation, Methodology, Project administration, Visualization, Writing – original draft. MG: Conceptualization, Formal analysis, Funding acquisition, Methodology, Supervision, Writing – review & editing. RH: Formal analysis, Methodology, Writing – review & editing. AB: Conceptualization, Funding acquisition, Project administration, Resources, Writing – review & editing. LO’G: Conceptualization, Formal analysis, Funding acquisition, Investigation, Methodology, Project administration, Supervision, Visualization, Writing – review & editing.
